# Systematic investigations on the reduction of 4-aryl-4-oxoesters to 1-aryl-1,4-butanediols with methanolic sodium borohydride

**DOI:** 10.3762/bjoc.6.94

**Published:** 2010-09-02

**Authors:** Subrata Kumar Chaudhuri, Manabendra Saha, Amit Saha, Sanjay Bhar

**Affiliations:** 1 Khorop High School, Howrah, West Bengal, India; 2 Department of Chemistry, Surendranath College (Evening), Kolkata 700 009, India; 3 Department of Chemistry, Organic Chemistry Section, Jadavpur University, Kolkata 700 032, India, Fax: +91-033-24146223

**Keywords:** diols, esters, lactones, reduction

## Abstract

4-Aryl-4-oxoesters undergo facile reduction of both the keto and the ester groups with methanolic NaBH_4_ at room temperature to yield the corresponding 1-aryl-1,4-butanediols whereas 4-alkyl-4-oxoesters furnish the corresponding 1,4-butanolides via selective reduction of the keto moiety. Results of a detailed and systematic investigation of the reaction are described.

## Introduction

Chemoselective reductions of aldehydes, ketones and imines are generally accomplished using NaBH_4_ in methanol where other reducible functional groups, e.g. esters, nitro, nitriles, etc., remain unaffected [1–10]. Although it has been reported that some aliphatic and aromatic esters have been reduced with a large excess of sodium or other metal borohydrides [11–12], often in higher boiling solvents [13] and in combination with various additives [14–15] including at a cationic micellar surface [16], selective reduction of the keto group in oxoesters has been accomplished using potassium borohydride in refluxing ethanol [17] where the product distribution critically depends on the relative proportions of substrate and reagent. Despite the occurrence of several recent reports of borohydride-mediated reduction of the ester moiety in α-oxo- [18–19] and β-oxoesters [20], sodium borohydride in various alcoholic solvents, often in the presence of additives [21], has been judiciously utilized [22] for the chemoselective reduction of the oxo-group, occasionally with subsequent transesterification and the formation of the alkoxy-modified β-hydroxyesters. γ-Oxoesters react chemoselectively with sodium borohydride to produce the corresponding γ-hydroxyesters [1–2,17,23–27] (sometimes in the form of γ-lactone) [24]. Following the above noted literature precedences [1–2,17,22–27] on the utility of NaBH_4_, we attempted to reduce 4-aryl-4-oxoesters with methanolic NaBH_4_ chemoselectively. Surprisingly, we found that 4-aryl-4-oxoesters underwent facile reduction of both the keto and the ester groups with methanolic NaBH_4_ at room temperature to yield the corresponding 1-aryl-1,4-butanediols whereas 4-alkyl-4-oxoesters furnished the corresponding 1,4-butanolides via selective reduction of the keto moiety. These results, to the best of our knowledge, have no literature precedence. We describe herein our systematic investigations to elucidate the different parameters involved in these reactions and to establish their synthetic usefulness.

## Results and Discussion

**Scheme 1:**
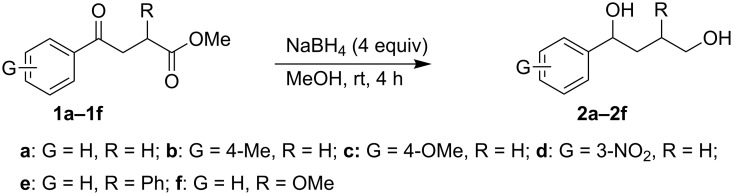
Facile reduction of γ-aryl-γ-ketoesters to the corresponding diols with methanolic NaBH_4_ at room temperature.

When, the γ-aryl-γ-ketoesters (**1a–1f**) were treated with methanolic NaBH_4_ (4 equiv) at room temperature (room temperature implies 30 °C throughout) both the oxo- and the alkoxycarbonyl moieties were reduced to give the diols (**2a–2f)**, as shown in Scheme 1.

**Scheme 2:**
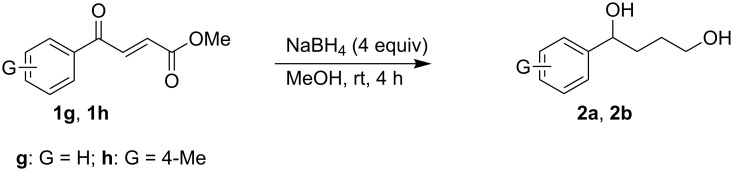
Facile reduction of γ-aryl-α,β-unsaturated-γ-ketoesters to the diols with methanolic NaBH_4_ at room temperature.

γ-Aryl-α,β-unsaturated-γ-ketoesters (**1g** and **1h**), on similar treatment, furnished the saturated diols (**2a** and **2b**) by the reduction of both the keto and the ester groups along with complete hydrogenation of the double bond (Scheme 2).

Detailed results are shown in Table 1.

**Table 1:** Reduction of 4-aryl-4-oxoesters (saturated and α,β-unsaturated) with NaBH_4_ in MeOH at room temperature (30 °C).

Entry	Substrate	Product	Yield (%)^a^

1	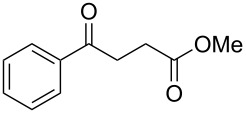 **1a**	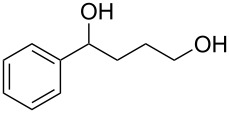 **2a**	82 [28–29]
2	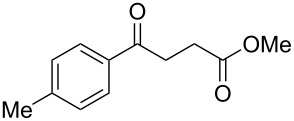 **1b**	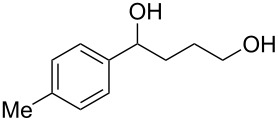 **2b**	86 [30]
3	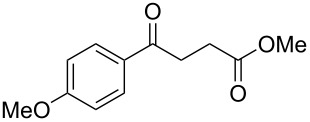 **1c**	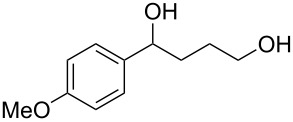 **2c**	80 [31–32]
4	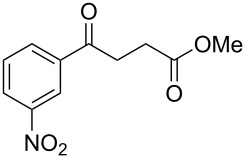 **1d**	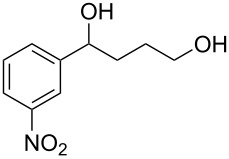 **2d**	71
5	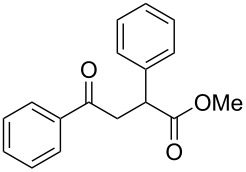 **1e**	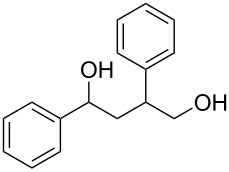 **2e**	67 [33]
6	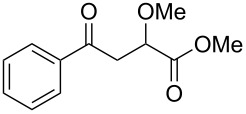 **1f**	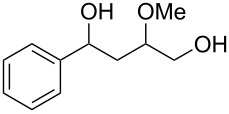 **2f**	81
7	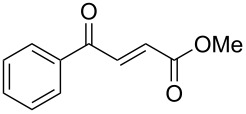 **1g**	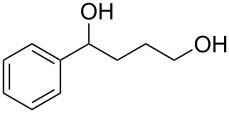 **2a**	83 [28–29]
8	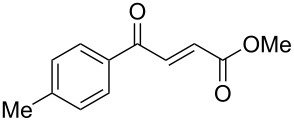 **1h**	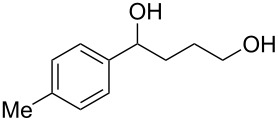 **2b**	85 [30]

^a^Yields refer to pure products, fully characterized spectroscopically (^1^H NMR, 300 MHz). References for known compounds are given in parenthesis after the respective yields.

**Scheme 3:**
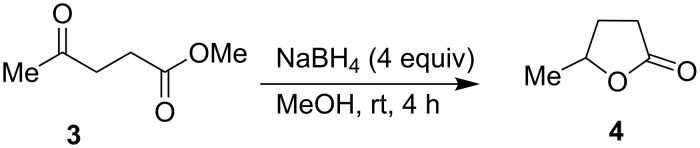
Facile reduction of γ-alkyl-γ-ketoester to the corresponding lactone with methanolic NaBH_4_ at room temperature.

At this point it is very interesting and important to note that only the oxo function of 4-alkyl-4-oxoester **3** was selectively reduced under the same conditions to yield lactone **4** without affecting the oxidation state of the alkoxycarbonyl moiety (Scheme 3).

From the results obtained so far, it is obvious that NaBH_4_ in methanol can be efficiently used for the synthesis of 1-aryl-1,4-butanediols from the easily accessible 4-aryl-4-oxoesters (Table 1) instead of employing the more costly and hazardous LiAlH_4_ which also often gives rise to several non-identifiable by-products. Structurally varied 1-aryl-1,4-butanediols are of great synthetic value with immense applications in cationic polymerizations [34], as intermediates for the syntheses of important acyclic antiviral nucleosides [35] and cyclic ethers [36].

**Scheme 4:**
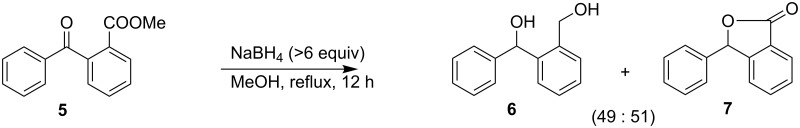
Reduction of methyl *o*-benzoylbenzoate with methanolic NaBH_4_.

Substrate **5** also underwent similar transformation under more drastic conditions to give a mixture of diol **6** [37] and lactone **7** [38], as shown in Scheme 4. In this instance no reaction took place at room temperature even after 24 h which might be ascribed to the lower electrophilicity of both the oxo- and alkoxycarbonyl functionalities of **5** from both electronic and steric standpoints.

**Scheme 5:**
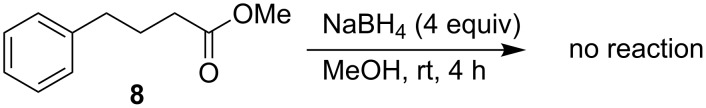
Reluctance of ester **8** towards reduction with methanolic NaBH_4_ at room temperature.

The *des*-keto ester **8**, as expected, was totally unaffected (Scheme 5) and was recovered unchanged.

Therefore, it is clear that the presence of both the aryl moiety and the oxo-function at the γ-carbon with respect to ester functionality is essential to bring about reduction of ester group with NaBH_4_. No reduction occurred when the reactions were carried out in anhydrous ether in place of methanol, however, substrates **1a** and **1b** in the ethereal medium underwent transformations in the presence of various protic polar co-solvents with different product distributions depending upon the nature of the co-solvent (Table 2).

**Table 2:** Reactions^a^ of **1a** and **1b** with NaBH_4_ in anhydrous ether in the presence of protic polar co-solvents.

Entry	SM	Co-solvent	Relative product distribution (%)^b^			
			Substrate	Lactone	Diol	Hydroxyester

1	**1a**	MeOH	–	37.1	62.9	–
2	**1a**	EtOH	–	40.6	59.4	–
3	**1a**	*t*-BuOH	5.8	94.2	–	–
4	**1a**	H_2_O	86.1	2.1	11.8	–
5	**1a**	AcOH	87.3	3.1	9.6	–
6	**1b**	MeOH	–	Trace	99.0	–
7	**1b**	EtOH	–	48.2	51.8	–
8	**1b**	*t*-BuOH	60.4	18.1	–	21.5
9	**1b**	H_2_O	48.7	15.4	–	35.9
10	**1b**	AcOH	21.9	51.4	–	26.6

^a^NaBH_4_ (4 equiv) in Et_2_O, co-solvent (2 equiv), 30 °C, 4 h. ^b^Determined by 300 MHz ^1^H NMR.

Compounds **1a**, **3**, acetophenone and butyrophenone were individually subjected to reduction in ether (Table 3) in the presence of MeOH (2 equiv) for a limited period of time (1 h). It was observed that the reduction of the keto group in the γ-oxoesters **1a** and **3** (entries 1 and 2 in Table 3) with the formation of the lactones **9** and **4** as one of the products was much faster than the reduction of aryl alkyl ketones (entries 3 and 4 in Table 3). Therefore, formation of lactone as the intermediate might be crucial for more facile reduction of the keto moiety in case of γ-oxoesters (entries 1 and 2 in Table 3), which is not possible in the case of normal aryl alkyl ketones (entries 3 and 4 in Table 3). It is also interesting to note that although in both **1a** and **3** the keto group was completely reduced, the relative proportion of the lactone (compared to hydroxyester) was much higher for **1a** than for **3**.

**Table 3:** Comparative study^a^ on reduction of various oxo-groups.

Entry	Substrate	Relative proportion (%)^b^ of		
		Substrate	Reduced products	
			Lactone	γ-Hydroxyester

1	**1a**	–	62.5	37.5
2	**3**	–	32.1	67.9
3	Acetophenone	49.2	50.8	
4	Butyrophenone	61.0	39.0	

^a^NaBH_4_ (4 equiv) in Et_2_O, MeOH (2 equiv), 30 °C, 1 h. ^b^Determined by 300 MHz ^1^H NMR.

**Scheme 6:**
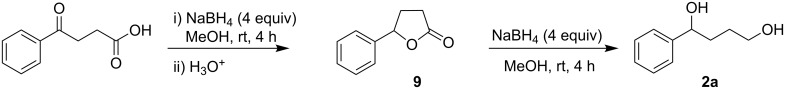
Intermediacy of a lactone in the formation of diol.

The intermediacy of lactone **9** [24] was also established by an independent route as outlined in Scheme 6.

**Scheme 7:**
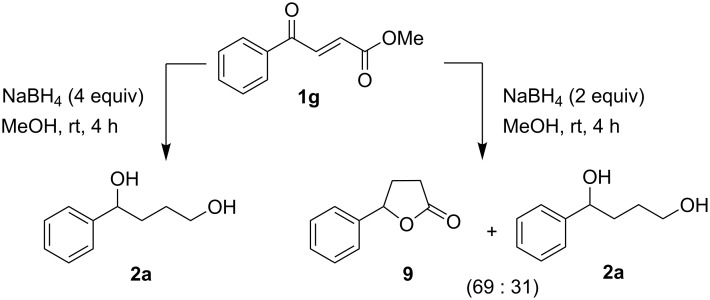
Diol formation from γ-aryl-α,β-unsaturated-γ-ketoester through the intermediacy of a saturated lactone during the reduction with methanolic NaBH_4_.

In order to prove the essentiality of the intermediacy of a lactone, compound **1g** (with the keto and ester moieties kept far apart for lactonization due to *trans*-geometry of the olefinic linkage) was treated with NaBH_4_ (4 equiv) in methanol. However, this reaction unexpectedly led to the exclusive formation of **2a**. With a smaller amount (2 equiv) of NaBH_4_ in methanol, compound **1g** gave **9** and **2a** in a ratio of 69:31(Scheme 7).

**Figure 1:**
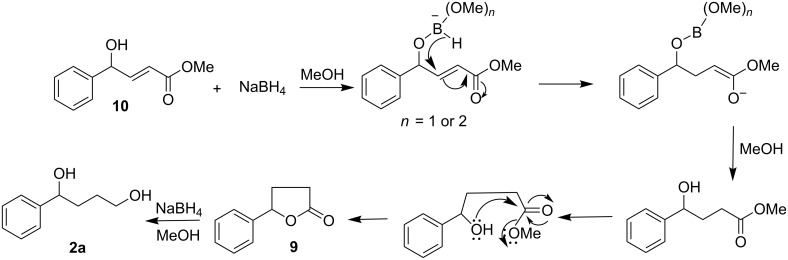
Mechanistic rationale for diol formation during the reduction of a γ-aryl-α,β-unsaturated-γ-ketoester with methanolic NaBH_4_.

It was presumed that the formation of **2a** from **1g** might occur through the initial reduction of the keto group with the formation of the γ-hydroxy-γ-aryl-α,β-unsaturated ester **10** [25]. In this connection it should be noted that when a limited amount of borohydride (1.2 equiv) was employed, we obtained the corresponding γ-hydroxy-*trans*-α,β-enoic ester **10** from **1g**. γ-Hydroxy-α,β-acetylenic esters have been reported [26] to undergo conjugate reduction of the triple bond with NaBH_4_ at low temperature (−34 °C) to give the corresponding γ-hydroxy-α,β-alkenoic esters, where the conjugate reduction does not proceed beyond the double bond. However, we have observed conjugate reduction of γ-hydroxy-α,β-alkenoic esters with methanolic NaBH_4_ (4 equiv) at 30 °C during the transformation of **10** to **2a**. Conjugate reduction here might be explained by the following plausible mechanistic scheme (Figure 1) where a mixed alkenyloxy alkoxy borohydride is initially formed by the reaction of **10** with sodium borohydride followed by conjugate reduction of olefinic linkage by intramolecular hydride attack to produce saturated 4-hydroxyester, which subsequently cyclizes to yield **9** and then further reduced to the diol **2a**.

**Scheme 8:**
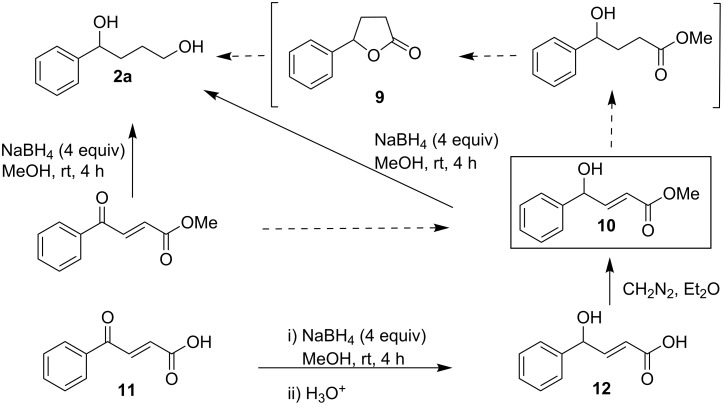
Intermediacy of γ-aryl-α,β-unsaturated-γ-hydroxyester during the reduction of γ-aryl-α,β-unsaturated-γ-ketoesters with methanolic NaBH_4_.

This postulate is supported by the observation that the proposed intermediate **10** (independently synthesized from **11**) is reduced to **2a** by the present method (Scheme 8, dotted arrows denote the route proposed in Figure 1).

The fact that the reduction of the keto group occurs before the conjugate reduction of the olefinic linkage has also been established in this study. In the basic reaction medium produced by NaBH_4_, the –COOH group is converted to –COO^−^, and as a result the double bond is no longer electron-deficient. The conjugate reduction by the intramolecular nucleophilic attack of the hydride is therefore not feasible. As a consequence, the –OH and –COO^−^ are too far apart to interact with each other. Therefore a single bond between the carbinol carbon and carboxylic acid moiety is impossible and hence no possibility of rotation, lactonization and subsequent reduction to diol **2a**. For this reason the γ-keto-α,β-enoic acid **11** on treatment with 4 equiv of NaBH_4_ in methanol smoothly furnished **12** as the preponderant product without conjugate reduction and subsequent reductive functional group transformation.

**Scheme 9:**
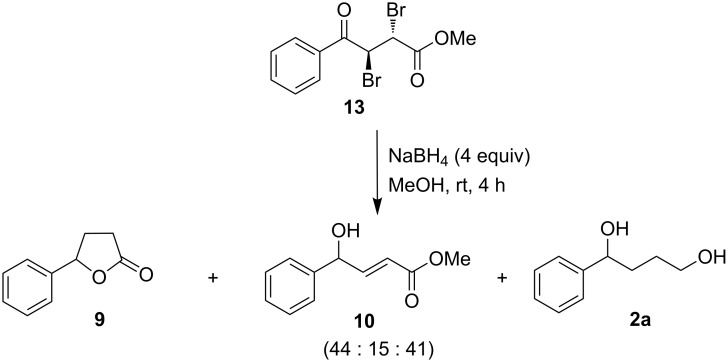
Reduction of γ-aryl-α,β-*anti*-dibromo-γ-ketoester with methanolic NaBH_4_.

When substrate **13** [39] (with *vicinal anti*-dibromo substituents to increase the rotational barrier of the single bond) was reacted with methanolic NaBH_4_ (4 equiv) at room temperature, a mixture of **9**, **10** and **2a** was obtained in a ratio of 44:15:41 (as determined by 300 MHz ^1^H NMR), as shown in Scheme 9.

**Scheme 10:**
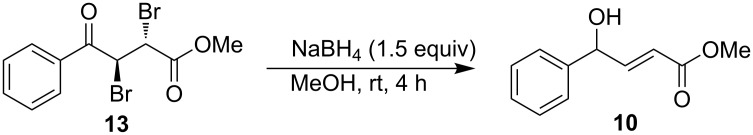
Intermediacy of γ-aryl-α,β-unsaturated-γ-hydroxyester during the reduction of γ-aryl-α,β-*anti*-dibromo-γ-ketoester with methanolic NaBH_4_.

Possibly, compound **13** was first reduced at the carbonyl function followed by concomitant dehydrobromination (under the basic reaction conditions), conjugate reduction at olefinic linkage, further dehydrobromination to **10** and subsequent conjugate reduction of **10** with the formation of **9** (as per the previous mechanistic scheme shown in Figure 1) and reduction of **9** to **2a**. The formation of **10** from **13** has been confirmed by the isolation of **10** (as the major product) as the outcome of the reaction of **13** with a limited amount of NaBH_4_ (1.5 equiv), as shown in Scheme 10.

**Scheme 11:**
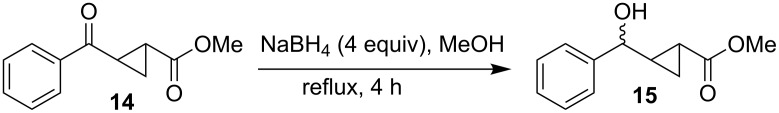
Chemoselective reduction of keto group in the presence of ester moiety where structural rigidity prevents the formation of a lactone intermediate during the reduction of γ-aryl-γ-ketoester with methanolic NaBH_4_.

The crucial role of the lactone formation during the borohydride-mediated reduction of 4-aryl-4-oxoester to 1,4-diols was finally established (Scheme 11) when substrate **14** [40] (incapable of lactonization due to distal spatial disposition of the oxo- and methoxycarbonyl moieties imposed by the rigidity of the cyclopropane ring system) underwent selective reduction of the oxo-functionality only under refluxing conditions to yield **15**. No significant reaction was observed at room temperature (monitored by TLC) even after 12 h.

From the investigations carried out so far, the intermediacy of a lactone during the NaBH_4_-mediated facile reduction of saturated and α,β-unsaturated-γ-aryl-γ-oxoesters to the corresponding saturated 1,4-butanediols has been firmly established. However, the reason for more facile reduction of the γ-aryl-lactones to diols and the relative reluctance of the γ-alkyl analogues is not yet clear.

## Conclusion

From the above study, a novel method utilizing NaBH_4_ in methanol that can provide clean, cost-effective and facile access to differently substituted 1-aryl-1,4-butanediols in good yield and high purity from the easily accessible precursors has been developed. The results also indicate that caution should be exercised when methanolic sodium borohydride is used as a reagent [1–2,17,22–27] for the chemoselective reduction of the keto group of all types of γ-oxoesters.

## Supporting Information

General experimental procedure for the NaBH_4_ reduction and the spectral data of the products are presented as supplementary data.

File 1 Experimental.
