# Supplementary material for: Systematic investigations on the reduction of 4-aryl-4-oxoesters to 1-aryl-1,4-butanediols with methanolic sodium borohydride
Source: Beilstein J Org Chem. 2010 Sep 2;6:748–55. doi: 10.3762/bjoc.6.94 (PMC2956461; doi:10.3762/bjoc.6.94)
Supplement: File 1 — Experimental. [file Beilstein_J_Org_Chem-06-748-s001.pdf]

Supporting information

to

Systematic investigations on the reduction of 4-aryl-4-oxoesters to 1-aryl-1,4-butanediols with methanolic sodium borohydride

Subrata Kumar Chaudhuri[1], Manabendra Saha[2], Amit Saha[3] and Sanjay Bhar*[3]

Address: [1]Khorop High School, Howrah, West Bengal, India; [2]Department of Chemistry, Surendranath College (Evening), Kolkata 700 009, India and [3]Department of Chemistry, Organic Chemistry Section, Jadavpur University, Kolkata 700 032, India, Fax: +91-033-24146223

Email: Sanjay Bhar - sanjaybharin@yahoo.com

* Corresponding author

Experimental

Representative procedure for the reduction of methyl 4-oxo-4-phenylbutanoate (1a) to 1-phenyl-1,4-butanediol (2a)

To a solution of methyl 4-oxo-4-phenylbutanoate (384 mg, 2 mmol) in anhydrous methanol (2.5 mL), was added sodium borohydride ($NaBH_4$, 304 mg, 8 mmol) in portions with constant stirring at 5–10 °C. After complete addition (within 5 min), the reaction mixture was allowed to reach room temperature (30 °C) and stirred for another 4 h at 30 °C until the reaction was complete (monitored by TLC). The methanol was removed under reduced pressure, the residue diluted

with water and extracted with dichloromethane (3 × 10 mL). The combined organic extracts were washed successively with water, then brine and dried over anhydrous Na_2SO_4. Evaporation of the solvent gave 1-phenyl-1,4-butanediol (**2a**) [1,2] which was further purified by filtration chromatography through a short-plug of silica gel with 10% ethyl acetate–petroleum ether as eluent to furnish pure **2a** (272 mg, 82%) as a colourless solid; mp 65–67 °C (from ether–hexane).

^{1}H NMR (300 MHz, $CDCl_3$): δ 1.67–1.73 (2H, m), 1.83–1.90 (2H, m), 2.02 (1H, br s), 3.68 (2H, dt, ^1J = 6.1 Hz, ^2J = 2.5 Hz), 4.73 (1H, t, J = 6.0 Hz), 7.25–7.35 (5H, m).

Spectral and physical data for the reduction products corresponding to Table 1 are given below.

1-(4-Methylphenyl)-1,4-butanediol [3] **(2b, entry 2)**

Low melting solid, yield 86%; purified by column filtration over silica gel using 10% ethyl acetate–hexane as eluent; colourless crystals from ether–hexane, mp 58–60 °C. ^{1}H NMR (300 MHz, $CDCl_3$): δ 1.56–1.71 (2H, m), 1.78–1.85 (2H, m), 2.32 (3H, s), 3.53–3.66 (2H, m), 4.62 (1H, t, J = 6.2 Hz), 7.12 (2H, d, J = 7.9 Hz), 7.20 (2H, d, J = 8.0 Hz).

1-(4-Methyoxyphenyl)-1,4-butanediol [4,5] **(2c, entry 3)**

Colourless highly viscous liquid, yield 80%; purified by column chromatography on silica gel using 6% ethyl acetate–hexane.

^{1}H NMR (300 MHz, $CDCl_3$): δ 1.74–2.29 (4H, m), 3.8 (3H, s), 3.83–3.93 (2H, m), 4.82 (1H, t, J = 7.1 Hz), 6.86 (2H, d, J = 8.7 Hz), 7.25 (2H, d, J = 8.7 Hz).

1-(3-Nitrophenyl)-1,4-butanediol (2d, entry 4)

Light yellow viscous liquid, yield 71%; purified by filtration through a small bed of silica gel using 6% ethyl acetate–hexane solution; sublimes at 130 °C/ 0.04 mmHg (with decomposition).

^{1}H NMR (300 MHz, $CDCl_3$): δ 1.69–1.97 (4H, m), 3.66–3.81 (2H, m), 4.86 (1H, distorted dd, ^1J = 7.7 Hz, ^2J = 4.1 Hz), 7.50 (1H, t, J = 7.9 Hz), 7.70 (1H, d, J = 7.6 Hz), 8.11 (1H, dd, ^1J = 8.1 Hz, ^2J = 0.9 Hz), 8.23 (1H, s).

Anal. Calcd for $C_{10}H_{13}NO_4$: C, 56.86; H, 6.20; N, 6.63. Found C, 56.74; H, 6.05; N, 6.78.

1,3-Diphenyl-1,4-butanediol [6] **(2e, entry 5)**

Colourless solid, mp 83 °C from dichloromethane–hexane yield 67%. ^{1}H NMR (300 MHz, $CDCl_3$): δ 1.90–1.99 and 2.08–2.24 (2H, m), 2.67–2.74 and 3.07–3.19 (1H, m), 3.66–3.76 (2H,

m), 4.52 (1H, dd, ^1J = 10.0 Hz, ^2J = 2.0 Hz), 4.60 (1H, t, J = 6.7 Hz) [for diastereomeric products], 7.13–7.42 (10H, m, aromatic-H).

^{13}C NMR (75 MHz, $CDCl_3$): δ 41.5, 43.0, 44.6, 46.2, 67.3, 67.8, 72.4, 72.6, 125.5, 126.1, 126.8, 126.9, 127.5, 127.7, 127.9, 128.0, 128.4, 128.5, 128.7, 142.0, 142.4, 143.91, 145.02.

3-Methoxy-1-phenyl-1,4-butanediol (2f, entry 6)

Highly viscous liquid, yield 81%; purified by column filtration on silica gel using 10% ethyl acetate–hexane as eluent.

^{1}H NMR (300 MHz, $CDCl_3$): δ 1.80–2.12 (2H, m), 3.41 (3H, s), 3.45–3.57 (1H, m), 3.75 (2H, dt, ^1J = 11.3 Hz, ^2J = 3.9 Hz), 4.85–4.91 (1H, m), 7.25–7.35 (5H, m).

^{13}C NMR (75 MHz, $CDCl_3$): δ 40.1, 40.4, 56.9, 57.4, 63.1, 63.5, 71.4, 72.2, 79.3, 80.3, 125.5, 125.7, 127.4, 127.5, 128.4, 144.4, 144.6.

Anal. Calcd for $C_{11}H_{16}O_3$: C, 67.32; H, 8.21. Found C, 67.89; H, 8.32.

Reaction of methyl 2-benzoylbenzoate (5) with $NaBH_4$

Methyl 2-benzoylbenzoate afforded the corresponding diol (**6**) [7] and lactone (benzofuranone, **7**) [8] in a 49:51 ratio (overall yield 60%) on treatment with excess (>6 equiv) $NaBH_4$ in refluxing MeOH for 12 h. No reaction took place at room temperature even after 24 h. The products were separated by column chromatography on silica gel using 15% ethyl acetate–petroleum ether as eluent. Spectral and physical data for compounds **6** and **7** are given below.

6: Colourless solid, mp 71–72 °C [7]; sublimes at 145–150 °C /0.4 mmHg.

^{1}H NMR (300 MHz, $CDCl_3$): δ 4.54 (2H, distorted AB quartet, J = 12.2 Hz), 6.02 (1H, s), 7.21–7.34 (9H, m).

7: Colourless solid, mp 112–113 °C from dichloromethane–petroleum ether; [8].

IR (neat): 1760 cm^{-1} ($\nu_{C=O}$ of lactone).

^{1}H NMR (300 MHz, $CDCl_3$): δ 6.40 (1H, s), 7.26–7.39 (5H, m), 7.55 (2H, t, J = 7.5 Hz), 7.65 (1H, dt, ^1J = 7.5 Hz, ^2J = 1.0 Hz), 7.96 (1H, d, J = 7.5 Hz).

Spectral and physical data for 4-methylbutan-1,4-olide [γ-methyl-γ-butyrolactone] (4) [9]

Colourless oil, yield 52%; purified by column filtration through a short bed of silica gel using 2% ethyl acetate–hexane as eluent; sublimes at 48–50 °C/ 1 mmHg.

IR (neat): 1772 cm^{-1} ($\nu_{C=O}$ of lactone).

^{1}H NMR (300 MHz, $CDCl_3$): δ 1.41 (3H, d, *J* = 6.3 Hz); 1.80–1.90 (2H, m); 2.31–2.45 (2H, m); 2.55 (2H, distorted t, *J* = 7.7 Hz); 4.59–4.68 (1H, m).

Preparation of methyl 4-oxo-4-phenyl-2,3-cyclopropanobutanoate (14) [10]

To a stirred mixture of phenacyl chloride (0.480 g, 3.14 mmol) in acetonitrile (12 mL), 1,4-diazabicyclo[2.2.2]octane (DABCO, 0.068 g, 0.6 mmol), Na_2CO_3 (0.410 g, 3.9 mmol) and methyl acrylate (0.4 mL, 4.5 mmol) were added and the reaction mixture was stirred at 80 °C for 20 h. The acetonitrile was removed under reduced pressure and the reaction product extracted with dichloromethane (3 × 15 mL), washed with water and dried (Na_2SO_4). Evaporation of the solvent gave crude **14**. Purification by column chromatography on silica gel using 6% ethyl acetate–petroleum ether as eluent furnished **14** as a gummy liquid (yield 0.395 g, 62%).

IR (neat): 1670 cm^{-1} ($\nu_{C=O}$ for aryl keto); 1730 cm^{-1} ($\nu_{C=O}$ for carbomethoxy).

^{1}H NMR (300 MHz, $CDCl_3$): δ 1.48–1.56 (2H, m), 2.29–2.35 (1H, m), 3.10–3.16 (1H, m), 3.64 (3H, s), 7.38 (2H, distorted t, *J* = 7.3 Hz), 7.49 (1H, t, *J* = 7.0 Hz), 7.93 (2H, d, *J* = 7.6 Hz).

Reduction of 14 with $NaBH_4$/methanol

Methyl 4-oxo-4-phenyl-2,3-cyclopropano-butanoate, **14** (250 mG, 1.2 mmol) was treated with $NaBH_4$ (184 mg, 4.8 mmol) in dry methanol (1.5 mL) with stirring at 30 °C for 4 h. No significant reaction was observed (monitored by TLC) after this time and even after 12 h. The reaction mixture was then refluxed for about 4 h and after the usual work-up, the crude product (oily residue) was obtained on evaporation of the solvent (CH_2Cl_2) under reduced pressure. Purification by filtration through a small bed of silica gel using 10% ethyl acetate–hexane gave methyl 4-hydroxy-4-phenyl-2,3-cyclopropanobutanoate, **15** (160 mg, 65%) as a highly viscous liquid.

IR (neat): 1720 cm^{-1} ($\nu_{C=O}$ of $-COOCH_3$); 3100–3600 cm^{-1}, broad (ν_{O-H}).

^{1}H NMR (300 MHz, $CDCl_3$): δ 0.98–1.25 (2H, m), 1.70–1.72 (1H, m), 1.80–1.86 (1H, m), 3.62 and 3.65 (s, for two diastereomeric alcohols, total 3*H*), 4.22 (d, *J* = 7.1 Hz) and 4.45 (d, *J* = 6.0 Hz) [total 1H for two diastereomeric alcohols].

References

1. Mori, N.; Omura, S.; Tsuzuki, Y. *Bull. Chem. Soc. Jpn.* **1965,** *38,* 1631–1634.
2. Tanner, D.; Groth, T. *Tetrahedron* **1977,** *53,* 16139–16146.
3. Bhat, K. S.; Rao, A. S. *Ind. J. Chem.* **1981,** *20B,* 355–358.
4. Mundryk, B.; Cohen, T. *J. Org. Chem.* **1989,** *54,* 5657–5659.
5. Kamal, A.; Sandbhor, M.; Shaik, A. A. *Tetrahedron Asymmetry* **2003,** *14,* 1575–1580.
6. Gurudutt, K. N.; Pasha, M. A.; Ravindranath, B.; Srinivas, P. *Tetrahedron* **1984,** *40,* 1629–1632.
7. Kirmse, W.; Kund, K. *J. Org. Chem.* **1990,** *55,* 2325–2332.
8. Newman, M. S. *J. Org. Chem.* **1961,** *26,* 2630–2633.
9. Karnik, A. V.; Patil; S. T.; Patnekar, S. S; Semwal, A. *New J. Chem.* **2004,** *28,* 1420–1422.
10. Papageorgiou, C. D.; Ley, S. V.; Gaunt, M. J. *Angew. Chem., Int. Ed. Engl.* **2003,** *42,* 828–830.
